# Where are the drugs? The scarcity of medications in the Western Pacific

**DOI:** 10.1016/j.lanwpc.2023.100728

**Published:** 2023-02-28

**Authors:** The Lancet Regional Health – Western Pacific

Many countries around the world are experiencing various drug shortages, which is affecting the quality of care being provided. These shortages are impacting multiple conditions, from acute infections to chronic diseases. As with many problems in the past few years, it would be convenient to blame these shortages on the COVID-19 pandemic. In the Western Pacific region, the recent pandemic-linked shortage was a scarcity of nirmatrelvir–ritonavir, a crucial antiviral drug used to treat COVID-19 in China. Demand spiked when COVID-19 policies were revised, and the country moved from a zero-COVID-19 principle to living with the disease. The sharp increase in COVID-19 cases and stalled discussions between the government and drug companies exposed the weakness of the supply chain. The increased demand was unmet, resulting in frantic illegal trades for antiviral medication. The outbreak in China also increased the need for fever-reducing medications, which consequently affected other countries in the Western Pacific region. Overseas-based family and friends of people in China sought out large quantities of antipyretic drugs, such as paracetamol, ibuprofen, and aspirin, to supplement the national crisis. In Singapore, customers stood in long queues outside courier companies to send medication to China, and pharmacies were sold out of essential pain relief. Similar situations were also observed in countries such as Japan and South Korea, where government warnings, purchasing limits, and price increases were implemented to manage the demand.

However, pharmaceutical supply issues have been a persistent challenge even in the pre-pandemic era, with publications on this issue increasing more than 13-fold between 2000 and 2020—a period now regarded as the drug shortage era. The consequences of COVID-19 have further exacerbated the situation, with shortages in Australia increasing by 300% following the pandemic's start. For instance, essential medicines are unavailable in Papua New Guinea, and the shortage is unrelated to COVID-19. 40% the population lives below the national poverty line, and the burden of purchasing medication for essential treatments is prohibitive to many. In many cases, health-care workers have been unable to treat people unless the patients could source their own privately funded medication. The situation is further compounded by a failure within the government to ensure the allocated budget is provided to the health-care system to acquire much-needed resources. As a result, the people of Papua New Guinea are facing a reduced quality of care and even some avoidable fatalities.

A multitude of other reasons, including peculiar ones such as social media trends, can also drive drug shortages. A recent social media fad has driven the current shortage of semaglutide, a drug used to manage type 2 diabetes by stimulating insulin production and treat obesity. The increased attention on platforms such as TikTok and Instagram has hailed the drug as a miracle weight loss solution. In Australia, increased demand for the off-label use of the semaglutide resulted in shortages that began in April, 2022, with supply levels not expected to normalise until April, 2023. Unfortunately, this has meant that many people with type 2 diabetes have faced increased stress in obtaining their medication or changing their pharmacological management.

The impact of these complex pharmaceutical shortages is multifaceted. Health-care providers have had to ration limited resources or find creative solutions in critical situations. Many patients need increased consultations with their health-care team to revise their management plans, which further burdens health-care systems that are already stretched to the brink. The increased human resources used to manage the shortage also lead to a more substantial economic burden on governments. The economic impacts also affect patients who face greater out-of-pocket costs in many instances, alongside the clinical implications for care. Drug shortages have been shown to cause an increase in errors, use of poor substitutions, and adverse events, including mortality.

Given these dire consequences of drug shortages, there is an urgent need to address this problem to reduce its impact. Prevention of drug shortages entirely is not practical: it requires a seamless supply line from production to delivery and accurate demand forecasting. Production requires adequate availability of raw materials, human resources, and infrastructure for production and delivery—all of which were grossly affected by the COVID-19 crisis. Procurement is the other aspect of ensuring adequate supply—poor forecasting of demand due to a sudden global pandemic or a social media fad can be unpredictable. The solutions often turned to in these cases, such as illegal trades and the use of telehealth pill mills, call for greater governmental oversight where policy regulation and clampdowns on illicit trade are much needed. However, instances of demand surge following policy shifts can and should be better prepared for, especially in countries dependent on external resources, such as Australia, which imports a large portion of its pharmaceutical cache.

These recent shortages are only a few examples of more substantial issues, but the consequences of an inadequate drug supply impact societies from an organisational level to each individual. There is no simple solution, but it calls for greater responsibility. A greater responsibility in governance to ensure adequate funding and access; greater accountability of organisations to set purchasing limits and increase community education in the face of shortages; greater responsibility from health-care providers for mindful prescribing; and greater responsibility in communities to prioritise the essential needs of the population.

